# Exploration of the optimal strategy for dietary calcium intervention against the toxicity of liver and kidney induced by cadmium in mice: An in vivo diet intervention study

**DOI:** 10.1371/journal.pone.0250885

**Published:** 2021-05-11

**Authors:** Zhaofang Chen, Kexin Shi, Wenjie Kuang, Lei Huang

**Affiliations:** 1 State Key Laboratory of Pollution Control & Resource Reuse, School of the Environment, Nanjing University, Nanjing, PR China; 2 Lamont-Doherty Earth Observatory, Columbia University, Palisades, NY, United States of America; Kafrelsheikh University, EGYPT

## Abstract

Cadmium (Cd) is a toxic non-essential element, while calcium (Ca) is an essential element with high chemical similarity to Cd. Dietary intake is the major Cd exposure pathway for non-smokers. A multi-concentration dietary intervention experiment was designed to explore the optimum concentration of Ca in diet with obvious protective effects against the toxicity of livers and kidneys induced by Cd in mice. The mice were divided into six groups with different concentrations of Cd and Ca in their food: control-group (no Cd or Ca), Ca-group (100 g/kg Ca, without Cd), Cd-group (2 mg/kg Cd, without Ca), Ca_L_+Cd-group (2 mg/kg Cd, 2 g/kg Ca), Ca_M_+Cd-group (2 mg/kg Cd, 20 g/kg Ca) and Ca_H_+Cd-group (2 mg/kg Cd, 100 g/kg Ca). The organ indexes, oxidative stress biomarkers, lesions and Cd concentrations were detected after a 30-day exposure period. Results showed that serum Aspartate Aminotransferase (AST) level in Ca_H_+Cd-group was significantly lower than that in Cd-group, while close to that in control-group. The contents of Serum Blood Urea Nitrogen (BUN) in different groups showed the same trend. Concentrations of all oxidative stress biomarkers (GSH-Px, SOD, CAT, GSH and MDA) in Ca_H_+Cd-group were close to the normal levels of control-group while significantly different from those in Cd-group. The only exception was the Malondialdehyde (MDA) levels in kidneys. This study suggests that Ca plays a protective role in relieving the Cd-induced toxicity of livers and kidneys and a concentration of 100 g/kg for Ca in diet showed the best protective effects. These findings could provide a clue for further studies concerning human diet intervention for Cd control.

## Introduction

Cadmium (Cd) is a serious toxic heavy metal derived mainly from industrial sources such as mining and smelting [1, 2]. Due to the rapid development of industrialization and urbanization, China has been suffering from severe heavy metal contamination in the past 20 years [3]. A national survey in 2018 showed that 19.4% of agricultural soils in China were contaminated by heavy metals [3], among which Cd played a major role [4]. Cd concentration in food like rice, wheat and vegetables has dramatically increased due to the influences of human activities and/or other planting activities on agricultural soils in China [5]. In up to 65% of rice from south China, the Cd concentration has exceeded the rice Cd standard of 0.20 μg/g used in China [6]. Dietary intake is the main route of Cd exposure for non-smokers [7, 8]. Considerable amount of Chinese may be exposed to Cd levels beyond the acceptable limits due to rice consumption [4]. From 1990 to 2015, the average dietary Cd intake for Chinese has been more than doubled, reflecting the increased risk of Cd pollution in the food chain [9]. With its long half-life, Cd could accumulate in organs and cause adverse health effects after entering human body [10]. Previous studies have further shown that Cd can cause toxic effects on the kidneys, livers, lungs, brain and testes, inducing osteoporosis, cardiovascular disease and even cancer [11–13].

Diet intake is an important source of Cd and of nutrients that could influence the absorption of Cd [14]. Previous studies have found that nutritional status could affect the accumulation and toxicity of Cd. It has been reported that several elements such as calcium (Ca), zinc (Zn), selenium (Se) and iron (Fe) may play a significant role in protecting humans and animals from Cd toxicity [15–17]. Ca is one of the essential elements for humans and performs a wide range of functions [18]. Due to the high chemical similarity to Cd [19], Ca has confirmed effects in regulating physiological or metabolic changes induced by Cd [20]. A recent study has found that 150 ~ 5000 mg/kg of Ca intake could effectively reduce relative bioavailability of rice Cd from 31 ~ 80% to 8.5 ~ 29% [2]. It has also been reported that the supplement of Ca could reduce its the absorption of Cd in intestine and therefore decrease the oxidative stress caused by Cd in rats. In comparison, Ca deficiency could increase the synthesis of Ca binding protein, resulting in the increase of intestinal absorption of Cd [21]. However, inadequate Ca intake has been a widespread nutritional problem across China. Data from the 2015 China Nutritional Transition Cohort Study showed that dietary Ca intake was insufficient for 94.3% people [22]. In summary, large amounts of Cd exposure and insufficient Ca intake may jointly exacerbate the threat caused by Cd, a thorny problem for public health in China.

Researches on the toxicity of Cd exposure as well as the interaction between Ca and Cd have been carried out worldwide [23, 24]. However, few studies have contained *in vivo* experiments simulating human dietary exposure pathways to explore the optimal Ca intervention concentration. We carried out a gradient Ca diet supplement strategy to explore the protective effects of Ca on organ damage induced by Cd. Our aim is to determine an optimal Ca concentration, which will provide the data support for further studies on reducing the risk of Cd to human health.

## Materials and methods

### Animals

A total of 36 5-week-old SPF (Specific Pathogen Free) Balb/c mice (half male and half female), weighing approximately 14.74 ± 1.21 g were purchased from Qinglongshan Experimental Animal Breeding Farm (Nanjing, China). These mice were acclimated for one week before experiment. During the acclimation period, the mice were fed with adequate Milli-Q water and mouse food which meets the standard of *Laboratory Animals–Nutrients for Formula Feeds* (GB 14924.3–2010) under standard animal room condition (12h:12h light/dark circulation, 25°C and 50% humidity) [25]. Animal feeding conformed to the Guide for the Care and Use of Laboratory Animal at Nanjing University and all the experiments with animals were carried out according to the guidelines of the institutional animal ethical committee. Experimental Animal Welfare Ethics Committee, Jiangsu Provincial Center for Disease Control and Prevention has reviewed the study protocol and approved this research (approval number: JSJK/JL-161). Some measures haven been taken to ensure the welfare of experimental animals. Before the experiment, the mice were acclimated for one week. During the experiment period, all the mice lived in the standard animal room and were fed with sufficient Milli-Q water and food. At the end of the experiment, the mice were euthanized using cervical vertebra dislocation to avoid long-term pain.

### Animal treatments

After one-week acclimation period, the mice were randomly divided into 6 groups with 6 mice in each group. In order to be consistent with the ratio of male to female in the natural environment, we set each group of male and female mice to be half each. There was no significant difference in body weights among the six groups at the beginning of the study. The female and male mice of each group were kept separately in two cages. We have already completed an experiment before, in which four groups of mice were fed mouse food containing different concentrations of Cd (S1 Table). According to the results of the experiment (S2 Table and S1–S7 Figs) and *National Food Safety Standard—Limit of Pollutants in Food* (GB 2762–2017), we chose 2 mg/kg Cd in diet as the dose of Cd exposure, which could induce significant toxicity of mouse organs compared with the control group within one-month exposure period. The groups included a control-group (without Cd or Ca in the mouse food), a Ca-group (with 100 g/kg Ca), a Cd-group (with 2 mg/kg Cd) and three Ca intervention groups. Considering the palatability of mouse food, the Ca concentration gradient is set as 2 g/kg, 20 g/kg and 100 g/kg. In addition to 2 mg/kg Cd, 2 g/kg, 20 g/kg and 100 g/kg Ca was added in the mouse food of the Ca_L_+Cd-group, Ca_M_+Cd-group and Ca_H_+Cd-group, respectively. All the mouse food was specially produced by the professional manufacturer (Suzhou Shuangshi Experimental Animal Feed Technology Co., Ltd.) according to the experimental design. Cd and Ca was added in the specially designed mouse food in the form of cadmium chloride (AR grade, Wanqinghua Glass Instrument Co., Ltd., Nanjing, China) and calcium carbonate (AR grade, Wanqinghua Glass Instrument Co., Ltd., Nanjing, China), respectively. In the 30-day exposure period, each mouse received approximately 4 g of specially-made feed at 9 a.m. every day and their weights were recorded. We monitored all the mice every day to confirm their growth status. No mice died during the experimental period. In the end of the exposure period, after fasting for 12 hours, each mouse was placed in a box containing 3% isoflurane for about 2–3 minutes until it was completely anesthetized. Then blood samples of mice were drawn from orbit and all the mice were euthanized using cervical vertebra dislocation.

### Sample preparation

The blood samples were centrifuged at 3500 r/min for 30 minutes after standing at room temperature for 2 hours. The serum was collected and stored in a -80°C freezer. The livers and kidneys were separated quickly and weighed the wet weights. One kidney was fixed in 4% paraformaldehyde solution for HE staining; another kidney was divided into two parts and weighed respectively. One part of kidney was immediately stored in -80°C for determination of Cd concentration. The other part of kidney was added with normal saline with the ratio of weight (g): volume(mL) = 1: 9, mechanically homogenized under ice water bath conditions and centrifuged at 2500 r/min for 10 minutes. The supernatants were taken and stored at a -20°C freezer for the determination of functional indicators and oxidative stress indicators. The liver of each mouse was evenly divided into three parts, and then the above procedure was repeated.

### The calculation of organ indexes

The organ indexes were calculated according to the following equation:

$$\text{Organ}\;\text{index}\;\left(\%\right)=\frac{\text{m}\;(\text{liver}\;\text{or}\;\text{kidney})}{\text{m}\;(\text{body})}\times 100\%$$ (1)

where m (liver or kidney) is the weight of the liver or the kidneys, m (body) is the body weight of the mouse.

### Serum biochemical assay

The serum Alanine Aminotransferase (ALT) and Aspartate Aminotransferase (AST) were measured using Alanine Aminotransferase assay kit (C009-2-1, Jiancheng Bioengineering Institute, Nanjing, China) and Aspartate Aminotransferase assay kit (C010-1-1, Jiancheng Bioengineering Institute, Nanjing, China), respectively. The Blood Urea Nitrogen (BUN) and Creatinine (Cr) were determined using Urea Assay Kit (C013-2-1, Jiancheng Bioengineering Institute, Nanjing, China) and Creatinine assay kit (C011-1-1, Jiancheng Bioengineering Institute, Nanjing, China), respectively. All the detection was conducted in Jiancheng Bioengineering Institute, Nanjing, China according to the manufacturer’s recommended protocol.

### Measurement of oxidative biochemical parameters in organs

The activities of Superoxide Dismutase (SOD), Glutathione Peroxidase (GSH-Px) and Catalase (CAT), as well as the levels of Malondialdehyde (MDA) and Glutathione (GSH) in livers and kidneys were determined in Jiancheng Bioengineering Institute, Nanjing, China. The above detection was conduction using SOD assay kit (A001-3-2, Jiancheng Bioengineering Institute, Nanjing, China), GSH-Px assay kit (A005-1-1, Jiancheng Bioengineering Institute, Nanjing, China), CAT assay kit (A007-1-1, Jiancheng Bioengineering Institute, Nanjing, China), MDA assay kit (A003-1-2, Jiancheng Bioengineering Institute, Nanjing, China) and GSH assay kit (A006-1-1, Jiancheng Bioengineering Institute, Nanjing, China), respectively according to the manufacturer’s recommended protocol.

### Histological examination

The livers and kidneys were fixed in 4% paraformaldehyde solution, embedded in paraffin, sliced into tissue slices and stained with Hematoxylin-Eosin (HE). The pathological structural changes were observed under an optical microscope (200×). The histological examination of liver and kidneys were recorded as 0 ~ 4 points based on the degree of lesions from light to heavy (normal—0 points, slight—0.5 points, mild—1 points, moderate—2 points, severe—3 points, very severe—4 points).

### Determination of Cd concentrations in organs

The samples of livers and kidneys were digested in 5 mL of 68% ultra-pure nitric acid over a night, then digested in a microwave digestion apparatus (Ethos UP, Milestone, Milan, Italy). The Cd concentrations in the livers and kidneys were measured by inductively coupled plasma mass spectrometry (ICP-MS, NexION 300X, PerkinElmer, Waltham, MA, USA.) with an indium isotope (114 In) as an internal standard. The determination was conducted according to the US Environmental Protection Agency (US EPA) Method 3050B. Reagent blank test was carried out before sample analysis to ensure that all reagent consumables were Cd interference-free. The limit of detection (0.045 μg/L and 0.068 μg/L for liver Cd detection and kidney Cd detection, respectively) was determined as the mean value plus three times of standard deviation of blank reagent, and half LOD was used for undetected samples. The Cd values of all the samples were higher than LOD.

### Statistical analysis

The values were expressed as mean ± standard deviation (SD) (n = 6). Statistical analysis was performed by SPSS software version 22.0 (IBM-SPSS Inc., Chicago, IL, USA) for windows. Two-way analysis of variance (ANOVA) with LSD post-hoc test was applied. Sex and treatment were set as two influencing factors. The results of the tests of between-subjects effects in different dependent variables were showed in S3 Table. Treatments showed the significant effect contributed to all the indicators except for SOD of kidney. Sexes showed the significant effect contributed to kidney index and MDA of kidney. An interaction effect of treatments and sexes contributed to BUN significantly. Therefore, the sex differences in the indicators were not considered in the results section. The relevant content was explained in the discussion section. All the graphics were drawn by GraphPad Prism software version 8.3.0. It was considered to be significant differences while the p value was less than 0.05.

## Results

### Organ index changes

**Fig 1:**
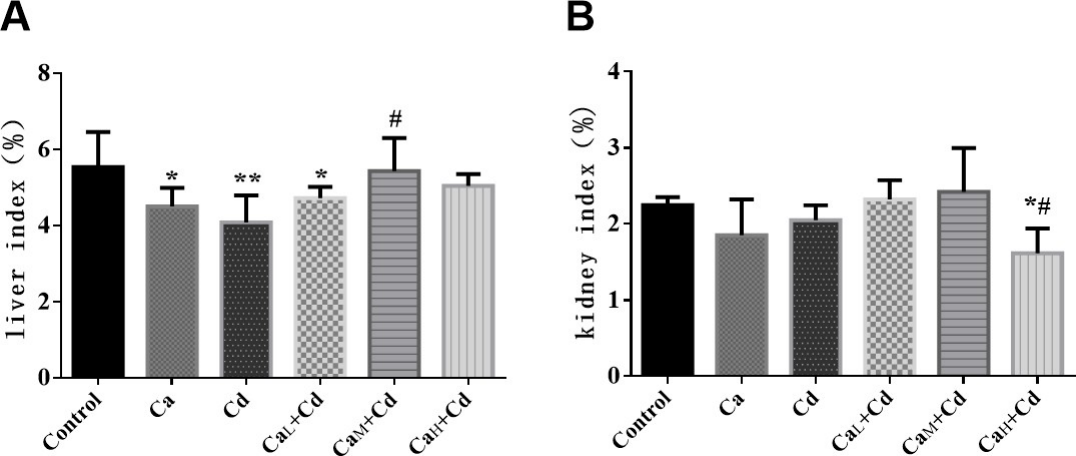
**The liver index (A) and kidney index (B) in different groups.** The data are shown as mean ± SD. ^*^ P < 0.05, compared with control-group. ^**^ P < 0.01, compared with control-group. ^**#**^ P < 0.05, compared with Cd-group. ^**##**^ P < 0.01, compared with Cd-group.

The liver index of Cd-group was significantly lower than that of control-group. It could be found that 20 g/kg Ca showed the best intervention effect, for the liver index in Ca_M_+Cd-group increased to the normal level of control-group, with significant difference from that of Cd-group (p < 0.05), while the intervention effects of Ca_L_+Cd-group and Ca_H_+Cd-group were not obvious (Fig 1A). As Fig 1B showed, the kidney index of Ca_H_+Cd-group was significantly lower than those of both control-group and Cd-group (p < 0.05). The two organ indexes of Ca group were both lower than those of control group, while the liver index of Ca group was significantly lower than that of control group.

### Changes of organ dysfunction

#### Serum ALT and AST of mice

**Fig 2:**
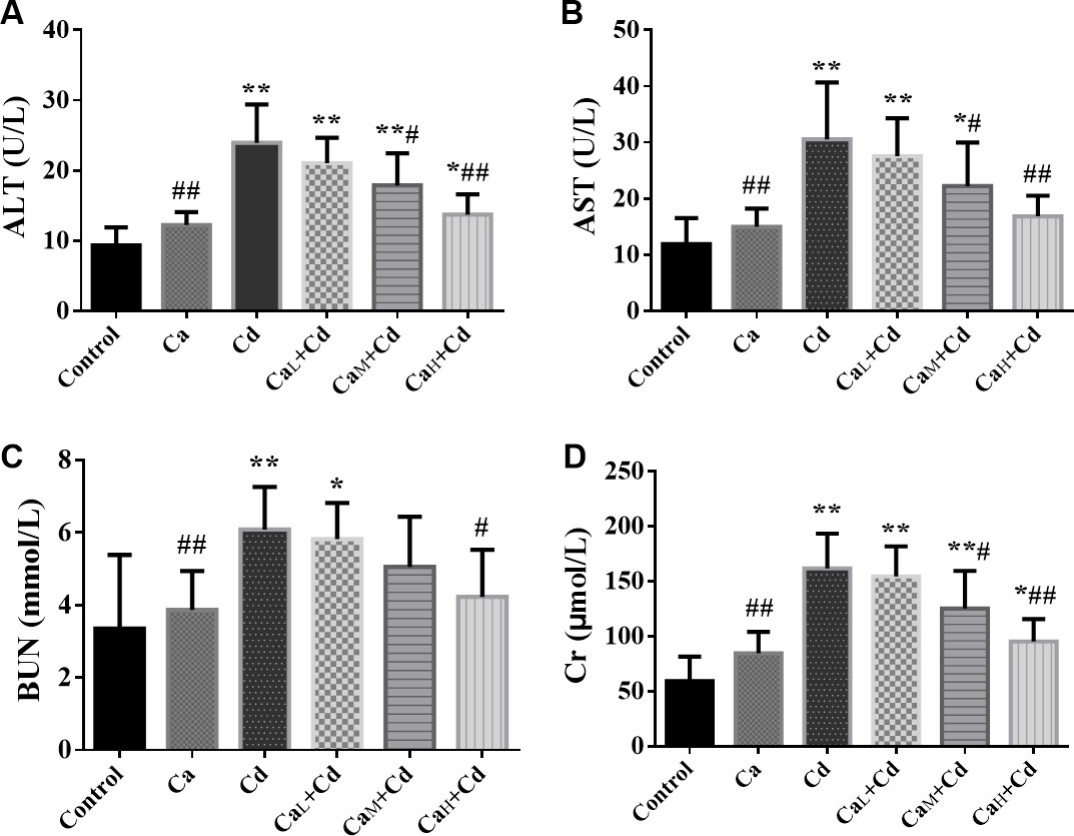
**The activity of serum ALT (A) and AST (B) and the concentration of serum BUN (C) and Cr (D) in different groups.** The data are shown as mean ± SD. ^*^ P < 0.05, compared with control-group. ^**^ P < 0.01, compared with control-group. ^**#**^ P < 0.05, compared with Cd-group. ^**##**^ P < 0.01, compared with Cd-group.

As Fig 2A and 2B showed, the serum ALT and AST activity of Cd-group was significantly higher than those of control-group (p < 0.05), indicating obvious liver dysfunction induced by Cd, while there was no significant difference between Ca-group and control-group. It can also be observed that Ca intervention could effectively decreased the serum ALT and AST activity, and the change was more obvious as the Ca concentration increased from 2 g/kg to 100 g/kg. The values of Ca_M_+Cd-group and Ca_H_+Cd-group were significantly lower than those of Cd-group, and 100 g/kg Ca showed the best protective effect on liver function.

#### Serum BUN and Cr of mice

The serum BUN (Fig 2C) and Cr (Fig 2D) of Cd-group were significantly higher than those of control-group (p < 0.05), while those of Ca-group showed no significant difference from control-group. The Ca intervention showed a relief to the kidney dysfunction induced by Cd-exposure, while the Ca_H_+Cd-group with high Ca concentration (100 g/kg) could effectively reduce the BUN concentration back to the normal level. As for Cr, Ca also showed an obvious intervention compared with Cd-group, although none of these three doses of Ca (2 g/kg; 20 g/kg; 100 g/kg) decreased the Cr concentration back to the normal level.

### Changes of oxidative stress in organs

#### Oxidative stress biomarkers of liver

**Fig 3:**
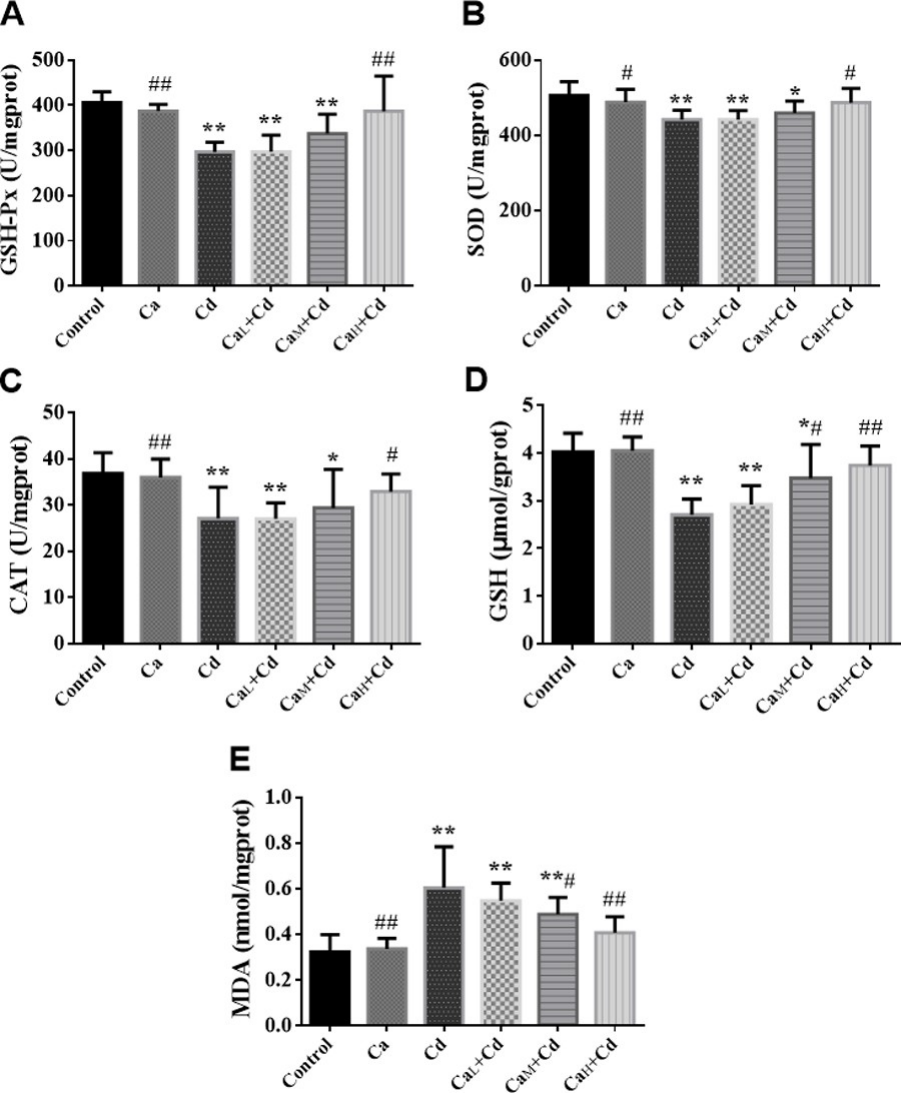
**The activity of GSH-Px (A), SOD (B) and CAT (C) and the content of GSH (D) and MDA (E) of liver in different groups.** The data are shown as mean ± SD. ^*^ P < 0.05, compared with control-group. ^**^ P < 0.01, compared with control-group. ^**#**^ P < 0.05, compared with Cd-group. ^**##**^ P < 0.01, compared with Cd-group.

It has been shown in Fig 3 that all the biomarkers in Ca-group kept on the normal level compared with control-group, while Cd significantly reduced the levels of GSH-Px (A), SOD (B), CAT (C) and GSH (D), meanwhile increased the level of MDA (E) in liver compared with those in control-group (p < 0.01). However, the administration of Ca effectively changed these indicators close to the normal levels, and the Ca_H_+Cd-group with high Ca concentration (100 g/kg) showed the best intervention effect, where the five indicators returned back to the normal levels.

#### Oxidative stress biomarkers of kidney

**Fig 4:**
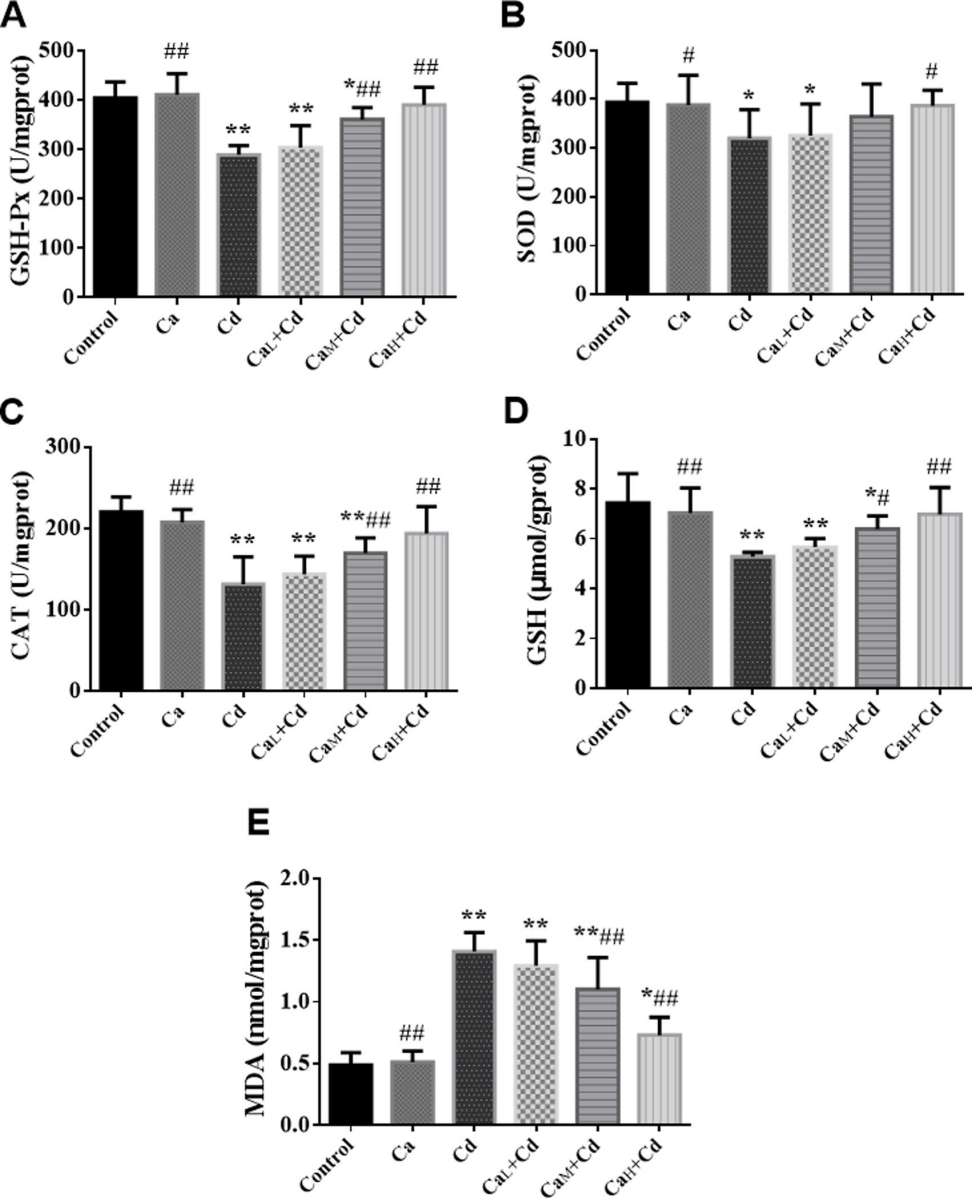
**The activity of GSH-Px (A), SOD (B) and CAT (C) and the content of GSH (D) and MDA (E) of kidney in different groups.** The data are shown as mean ± SD. ^*^ P < 0.05, compared with control-group. ^**^ P < 0.01, compared with control-group. ^**#**^ P < 0.05, compared with Cd-group. ^**##**^ P < 0.01, compared with Cd-group.

The change trend of the biomarkers in kidney was approximately similar with that in liver. It can be observed from Fig 4 that the biomarkers in Ca-group were at the same level of control-group, while Cd-group showed significant differences in all the biomarkers compared with control-group (p < 0.05). It has been shown that Ca could increase GSH-Px (A), SOD (B), CAT (C) and GSH (D) compared with Cd-group, and the indicators in Ca_H_+Cd-group (Ca 100 g/kg) increased back to the normal level. Meanwhile, MDA (E) content decreased as the Ca concentration increased, although it didn’t completely return to the normal level within the intervention of different Ca concentrations in this research.

### The histopathological analysis of organs

#### The pathological changes of liver

**Fig 5:**
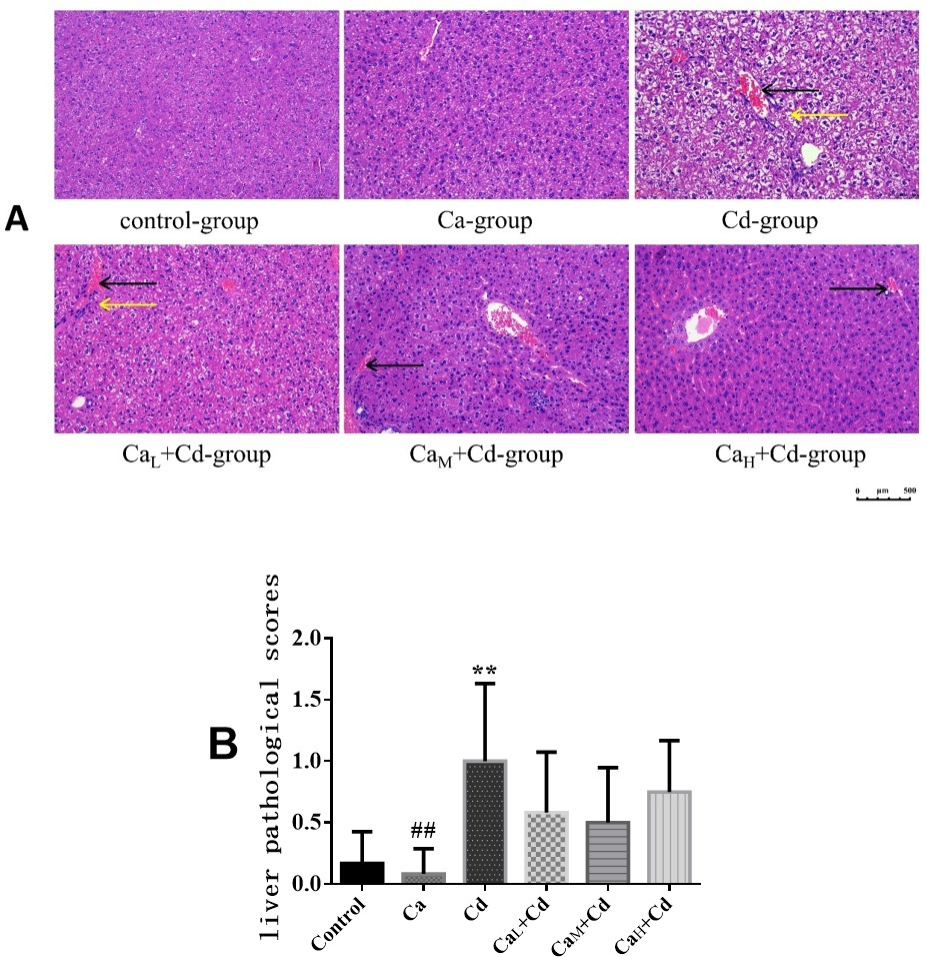
**The pathological sections (A) and pathological scores (B) of livers in different groups.** (A) Pathological sections of liver. Pictures were taken at 200× magnification and the bar indicates 500 μm. Yellow arrows indicate inflammatory cell infiltration, and black arrows indicate sinus congestion; (B) Liver pathological scores. The data are shown as mean ± SD. ^*^ P < 0.05, compared with control-group. ^**^ P < 0.01, compared with control-group. ^**#**^ P < 0.05, compared with Cd-group. ^**##**^ P < 0.01, compared with Cd-group.

As Fig 5A showed, the liver section images of control-group and Ca-group showed the normal appearance, without obvious pathological changes. However, obvious sinusoidal congestion and inflammatory cell infiltration could be observed in the liver section of Cd-group. The sinusoidal congestion and inflammatory cell infiltration in Ca_L_+Cd-group were reduced, and there wasn’t inflammatory cell infiltration symptom in Ca_M_+Cd-group and Ca_H_+Cd-group, indicating that administration of Ca could partly relieve the pathological changes induced by Cd.

It could be observed from Fig 5B that liver pathological score in Ca-group was at the normal level and even lower than that in control-group, while the mean liver pathological score in Cd-group was 5.99 times that of control-group, showing a significant difference (p < 0.01). The groups with Ca-intervention showed the lower pathological scores compared with Cd-group, indicating effects of Ca on relieving liver pathological changes induced by Cd, however, there were no significant differences between Ca-intervention groups and Cd-group.

#### The pathological changes of kidneys

**Fig 6:**
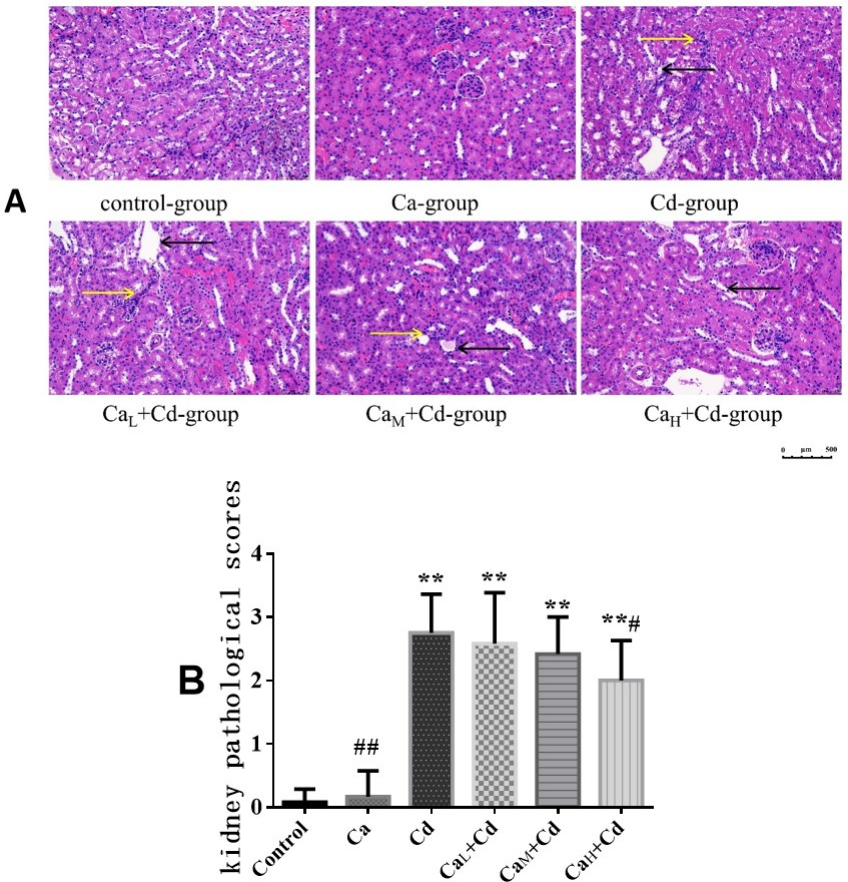
**The pathological sections (A) and pathological scores (B) of kidneys in different groups.** (A) Pathological sections of kidneys. Pictures were taken at 200× magnification and the bar indicates 500 μm. Yellow arrows indicate inflammatory cell infiltration, and black arrows indicate transparent casts. (B) Kidney pathological scores. The data are shown as mean ± SD. ^*^ P < 0.05, compared with control-group. ^**^ P < 0.01, compared with control-group. ^**#**^ P < 0.05, compared with Cd-group. ^**##**^ P < 0.01, compared with Cd-group.

As Fig 6A showed, there were no obvious pathological changes in control-group and Ca-group, while infiltration of inflammatory cells and transparent casts were observed in Cd-group. Meanwhile, the symptoms in Ca intervention groups were relieved as the Ca concentrations increased, which indicated that Ca could partly relieve the renal pathological changes although the symptoms couldn’t disappear completely.

The kidney pathological scores in Fig 6B showed a similar trend with the pathological section images. The kidney pathological score in Ca-group (0.167 ± 0.408) was slightly higher than that in control-group (0.08 ± 0.20), both in a low level and without significant difference, while the mean score in Cd-group (2.75 ± 0.61) was approximately 33 times that of control-group (0.08 ± 0.20), indicating significant effect of Cd on kidney pathological changes (p < 0.01). The kidney pathological scores decreased while adding Ca in the mice diet, and the scores were lower as the Ca concentration increased, however, even the highest Ca concentration (100 g/kg) didn’t make the damage return to the normal levels.

### Cd contents of organs

**Fig 7:**
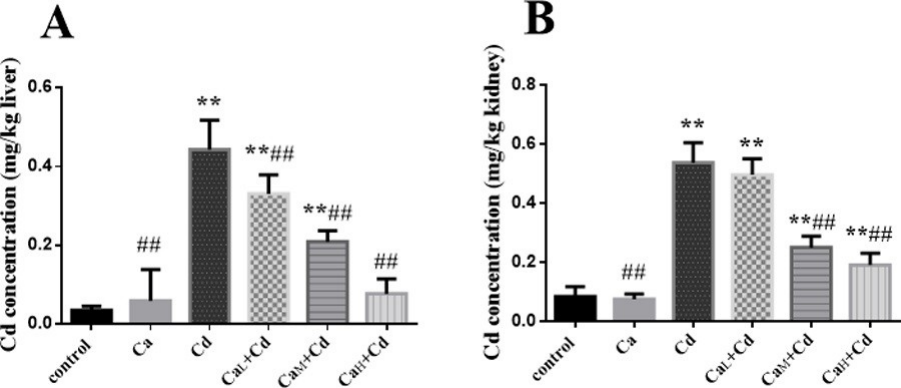
**The Cd concentrations in liver(A) and kidney(B).** The data are shown as mean ± SD. * P < 0.05, compared with control-group. ** P < 0.01, compared with control-group. ^#^ P < 0.05, compared with Cd-group. ^##^ P < 0.01, compared with Cd-group.

As showed in Fig 7A, Cd concentrations of liver in control-group and Ca-group were both at a low level (0.035 ± 0.010 mg/kg liver and 0.059 ± 0.079 mg/kg liver, respectively). The value in Cd-group (0.443 ± 0.074 mg/kg liver) was significantly higher than that in control-group (p < 0.01). The Ca intervention groups showed obvious decrease in Cd concentration compared with that in Cd-group, while the value in Ca_H_+Cd-group was decreased to the normal level, showing no significant difference from that in control-group.

The Cd concentration of kidney in Cd-group (0.538 ± 0.067 mg/kg kidney) was significantly higher than that in control-group (p < 0.01). The Ca_L_+Cd-group showed no significantly decrease in Cd concentration from that in Cd-group, while Cd concentrations in Ca_M_+Cd-group and Ca_H_+Cd-group were significantly lower than that in Cd-group (p < 0.01). However, even the Cd concentration in Ca_H_+Cd-group was still significantly higher than that in control-group (Fig 7B).

## Discussion

Prior studies have found the quite long half-life of Cd in human body. Once absorbed, Cd will accumulate in the human body, especially in the livers and kidneys for a long time, which contains approximately 60% of the total Cd in the whole body [26]. It is partly because there is a large number of metallothionein in these organs, which is easy to bind with heavy metals such as Cd [27]. The toxicity to liver and/or kidneys of mice induced by Cd has been reported in previous studies, which is specifically manifested in liver and kidney dysfunction, oxidative stress and pathological changes [28–31]. Ca is known as an essential element that is of significance in whole-body growth [32]. It has been reported that the content of Ca in body can significantly influence Cd absorption in the gastrointestinal tract, for Cd use the same intestinal transporters as Ca in both animals and humans [17, 21]. With a high chemical similarity to Cd, Ca can regulate physiological or metabolic changes in organisms induced by Cd [33]. In our study, all indicators in Ca-group were not significantly different from those in control-group, which excluded the threat of Ca supplementation on the health of mice, whereas there was significant toxicity of liver and kidneys in mice exposed to Cd as compared with the mice in control-group. In comparison, Ca intervention effectively protected mice from organ dysfunction, oxidative damage and lesions induced by Cd, indicating protective effects of Ca on Cd-induced organ toxicity.

The ALT and AST in the blood were used as important indicators of clinical liver function, which will increase when liver function is impaired [34]. Serum Blood Urea Nitrogen (BUN) and Creatinine (Cr) levels are important indicators for evaluating glomerular filtration function and are often used in the diagnosis of acute kidney injury syndrome [35]. These indicators in Cd-group were all significantly higher than those in control-group (p < 0.01), suggesting obvious liver and kidney dysfunction induced by Cd, which was consistent with the results in prior studies [30, 31, 36]. Whereas, Ca showed protective effects on the organ dysfunction, especially in Ca_H_+Cd-group, the level of AST and BUN decreased to the normal level of control-group. The changes in lipid peroxide content and the activity of antioxidant enzymes are often used as biomarkers of oxidative stress caused by Cd to assess the degree of oxidative damage to cells [37]. In this study, the activities of the antioxidant enzymes SOD, CAT and GSH-Px and the levels of the non-enzymatic antioxidant GSH of liver and kidneys significantly decreased in Cd-group compared control-group, while the levels of MDA increased significantly (p < 0.05). The changes of SOD, CAT, GSH-Px and MDA were parallel to the results of several previous studies [30, 31, 36]. By contrast, these indicators in Ca intervention groups, especially Ca_H_+Cd-group, were reversed to the levels in control-group. It has been reported that Cd exposure increased the reactive oxygen species (ROS), and the over-production of ROS may be one of the causes for oxidative stress in liver and kidneys [30, 31, 36, 38]. As a significant Cd-chelator, GSH plays an important role in intracellular Cd detoxification [39]. The reduction in the activities of these antioxidant enzymes and non-enzymatic antioxidant can weaken the ability of organisms to scavenge free radicals, leading to oxidative damage in organs [40]. The MDA is a metabolite of lipid peroxidation, which has been considered as an index of oxidative stress. It can be referred that Cd may inhibit the antioxidative system and cause overproduction of ROS, leading to the oxidative stress of organs. Based on the findings of previous studies [29, 31, 36, 41], the oxidative stress in liver further damaged the structural integrity of liver cell membranes, causing the leakage of ALT and AST into blood, which also explained the increase of serum ALT and AST induced by Cd. Antioxidant therapy has been considered as a significant approach to inhibiting Cd toxicity [30, 42]. It can be derived that Ca may indirectly protect organs from oxidative stress by increasing the content of GSH, and further protected organ function.

Meanwhile, histological examination in our study revealed that Cd induced liver and kidney pathological changes. In Cd-group, the pathological section of liver showed inflammatory cell infiltration and sinus congestion, while that of kidney showed infiltration of inflammatory cells and transparent casts. Although Ca intervention could partly relieve the pathological changes induced by Cd, even the highest concentration of Ca (100 g/kg) couldn’t completely eliminate Cd-induced lesions in liver and kidneys. The Cd concentrations in liver and kidneys of Cd-group were both significantly higher than those of control-group, which indicating higher Cd accumulation in organs because of higher Cd intake. Compared with Cd-group, the Cd concentrations in Ca intervention groups showed decrease trend with a dose-dependence.

Once absorbed by bodies, Cd is easy to bind to metallothionein and accumulate in target organs such as liver and kidney [24], which can explain the significantly higher concentrations of Cd in livers and kidneys in Cd-group compared with control-group.

It has been verified that the combination of cadmium and MT can reduce the toxicity of cadmium to mouse organs [43]. A previous study has found the MT concentration and the binding rate of Cd to MT in the group added Ca in advance were both higher than those in the group exposed to Cd without Ca [44]. Another study has found that the Cd accumulation in livers and kidneys in mice fed with Ca-deficient food was significantly higher in the mice fed with Ca-rich food after 6-week exposure period, and the accumulation might be strongly correlated with the mRNA expression of Ca transporter and metallothionein in intestine [24]. Based on previous studies and our results in this study, we inferred that it was partly because Ca increased MT concentration and further promoted the combination of MT and Cd, reducing the toxicity of Cd to livers and kidneys. On the other hand, Ca decreased Cd bioavailability for uptake at the mammalian gut epithelium, thus decreasing the absorption and accumulation of Cd in organs, which further protected livers and kidneys from the toxicity induced by Cd. However, the competition between Ca and Cd is limited and it is the dietary intake of Cd, rather than elimination which determines the amount of Cd accumulation in the body. Therefore, the supplement of Ca can partially reduce the Cd accumulation in the body, but it cannot completely eliminate the damage and lesions in livers and kidneys caused by Cd.

Generally speaking, Ca intervention decreased Cd accumulation, decreased the degree of organ dysfunction and oxidative stress in liver and kidneys induced by Cd, and the effect of intervention was in a dose-dependent manner. In this study, Ca_H_+Cd-group (Ca 100 g/kg) showed the best protective effects on both liver and kidney injury induced by Cd. It has been found that Ca deficiency could increase Cd uptake in enterocytes and further lead to higher Cd absorption [21], which is correspond with our results. On the other hand, due to the competition between Ca^2+^ ions and Cd^2+^ ions, Cd^2+^ ions will be easy to bind with calcium binding protein (CaBP) and transport in the blood stream in the condition of Ca deficiency [45], thus leading to the increase of Cd accumulation and the degree of toxicity in organs.

According to the results of the two-way analysis of variance (S3 Table), we found that only the kidney index and MDA of kidney showed significant sex differences. The kidney index of female mice was significantly lower than that of male mice (1.862 ± 0.377% vs.2.303 ± 0.438%, p<0.05). The MDA of kidney of female mice was significantly lower than that of male mice (0.856 ± 0.036 vs. 0.986 ± 0.036 nmol/mgprot, p < 0.05). A previous study has found that female mice showed higher Cd accumulation in the kidneys and liver. It was mainly due to the Cd accumulation regulated by sex hormones, e.g., estro-gen, progesterone, and testosterone [46]. It was showed in S8 Fig that the interaction effect of treatments and sexes contributed to BUN was significantly. However, such an interaction was extremely individual, we cannot analyze a clear mechanism from it.

In this study, we explored the effects of Cd exposure on mice from the aspects of dysfunction, oxidative stress and histopathology of liver and kidney. In addition, the protective effects of Ca on Cd toxicity in mice were explored to get an optimal ratio of Ca in diet that could decreased toxicity I nduced by Cd, which provided theoretical basis and data support for Cd pollution prevention. Furthermore, the results of this study are of great significance to formulate effective nutritional element intervention strategies to reduce human health risks caused by Cd. However, there are still some limitations in this study. The more samples and longer exposure period are needed to avoid uncertain gap in the future. In addition, we choose only one element Ca as the dietary supplement to explore the effects of Ca on the toxicity induced by Cd, which is not comprehensive. We will also try to investigate the protective effects of various nutrients on Cd-induced toxicity and determine an optimal concentration formula of several nutrients, which will provide strategies for reducing the Cd-induced health risks of humans in our future study.

## Conclusions

In conclusion, Ca displays protective effects on organ dysfunction, oxidative stress, pathological lesions as induced by Cd as well as reduce Cd accumulation in livers and kidneys. For mice, 100 g/kg was the optimal dietary concentration of Ca in intervention. These findings will provide references for further researches investigating the diet strategies to reduce the health risks of human Cd exposure. However, the fact that Ca supplement can’t completely eliminate the liver and kidney damages caused by Cd suggests that it is more important to reduce Cd intake than to provide nutrition supplement so as to fundamentally tackle problems related to Cd exposure and protect human health.

## Supporting information

S1 Fig The activity of serum ALT (A) and AST (B) in different groups.
(DOCX)

S2 Fig The concentration of serum BUN (C) and Cr (D) in different groups.
(DOCX)

S3 Fig The activity of GSH-Px, SOD and CAT and the content of GSH and MDA of liver in different groups. (DOCX)

S4 Fig The activity of GSH-Px, SOD and CAT and the content of GSH and MDA of kidney in different groups. (DOCX)

S5 Fig The pathological scores of liver and kidney in different groups. (DOCX)

S6 Fig The pathological sections of liver in different groups. (DOCX)

S7 Fig The pathological sections of kidney in different groups. (DOCX)

S8 Fig The interaction effect of treatments and sexes contributed to serum BUN. (DOCX)

S1 Table Dose of cadmium in the feed of different experimental groups. (DOCX)

S2 Table Liver and kidney indexes in different groups. (DOCX)

S3 Table The tests of between-subjects effects in different dependent variables. (DOCX)

S4 Table The descriptive statistics of the kidney index and MDA of kidney. (DOCX)

## Abbreviations used

Cd: cadmium
Ca: calcium
ALT: alanine aminotransferase
AST: aspartate aminotransferase
BUN: blood urea nitrogen
Cr: creatinine
GSH-Px: glutathione peroxidase
SOD: superoxide dismutase
CAT: catalase
GSH: glutathione
MDA: malondialdehyde
ROS: reactive oxygen species
HE: hematoxylin-eosin
ICP-MS: inductively coupled plasma mass spectrometry
LOD: limit of detection

## References

[1] Satarug S, Garrett SH, Sens MA, Sens DA. Cadmium, Environmental Exposure, and Health Outcomes. Environmental Health Perspectives. 2010;118(2):182–90. 10.1289/ehp.0901234 20123617 PMC2831915

[2] Zhao D, Juhasz AL, Luo J, Huang L, Luo X-S, Li H-B, et al. Mineral Dietary Supplement To Decrease Cadmium Relative Bioavailability in Rice Based on a Mouse Bioassay. Environmental Science & Technology. 2017;51(21):12123–30. 10.1021/acs.est.7b02993 28960068

[3] Delang CO. Heavy metal contamination of soils in China: standards, geographic distribution, and food safety considerations. A review. Erde. 2018;149(4):261–8.

[4] Zhao F-J, Ma Y, Zhu Y-G, Tang Z, McGrath SP. Soil Contamination in China: Current Status and Mitigation Strategies. Environmental Science & Technology. 2015;49(2):750–9. 10.1021/es5047099 25514502

[5] Hu Y, Cheng H, Tao S. The Challenges and Solutions for Cadmium-contaminated Rice in China: A Critical Review. Environment International. 2016;92–93:515–32. 10.1016/j.envint.2015.11.003 27179698

[6] Zhu P, Liang X-x, Wang P, Wang J, Gao Y-h, Hu S-g, et al. Assessment of dietary cadmium exposure: A cross-sectional study in rural areas of south China. Food Control. 2016;62:284–90.

[7] Nordberg GF, Bernard A, Diamond GL, Duffus JH, Illing P, Nordberg M, et al. Risk assessment of effects of cadmium on human health (IUPAC Technical Report). Pure and Applied Chemistry. 2018;90(4):755–808.

[8] Satarug S, Vesey DA, Gobe GC. Current health risk assessment practice for dietary cadmium: Data from different countries. Food and Chemical Toxicology. 2017;106:430–45. 10.1016/j.fct.2017.06.013 28602857

[9] Song Y, Wang Y, Mao W, Sui H, Yong L, Yang D, et al. Dietary cadmium exposure assessment among the Chinese population. Plos One. 2017;12(5). 10.1371/journal.pone.0177978 28542445 PMC5436861

[10] Wu X, Liang Y, Jin T, Ye T, Kong Q, Wang Z, et al. Renal effects evolution in a Chinese population after reduction of cadmium exposure in rice. Environmental Research. 2008;108(2):233–8. 10.1016/j.envres.2008.02.011 18692183

[11] Liang Y, Lei L, Nilsson J, Li H, Nordberg M, Bernard A, et al. Renal Function after Reduction in Cadmium Exposure: An 8-Year Follow-up of Residents in Cadmium-Polluted Areas. Environmental Health Perspectives. 2012;120(2):223–8. 10.1289/ehp.1103699 22027495 PMC3279438

[12] Murashima M, Kikuchi Y, Nomiyama T, Kumagai N, Omae K, Watanabe S, et al. Intake and excretion of cadmium and iron balance and influence of dietary habits among young women. Nihon eiseigaku zasshi Japanese journal of hygiene. 2004;59(1):31–7. 10.1265/jjh.59.31 15007902

[13] Nagata C, Nagao Y, Shibuya C, Kashiki Y, Shimizu H. Urinary cadmium and serum levels of estrogens and androgens in postmenopausal Japanese women. Cancer Epidemiology Biomarkers & Prevention. 2005;14(3):705–8. 10.1158/1055-9965.EPI-04-0619 15767353

[14] Burganowski R, Vahter M, Queirolo EI, Peregalli F, Baccino V, Barcia E, et al. A cross-sectional study of urinary cadmium concentrations in relation to dietary intakes in Uruguayan school children. Science of the Total Environment. 2019;658:1239–48. 10.1016/j.scitotenv.2018.12.220 30677986 PMC6369586

[15] El-Boshy ME, Risha EF, Abdelhamid FM, Mubarak MS, Ben Hadda T. Protective effects of selenium against cadmium induced hematological disturbances, immunosuppressive, oxidative stress and hepatorenal damage in rats. Journal of Trace Elements in Medicine and Biology. 2015;29:104–10. 10.1016/j.jtemb.2014.05.009 24954678

[16] Ognjanovic BI, Markovic SD, Pavlovic SZ, Zikic RV, Stajn AS, Saicic ZS. Effect of chronic cadmium exposure on antioxidant defense system in some tissues of rats: Protective effect of selenium. Physiological Research. 2008;57(3):403–11. 10.33549/physiolres.931197 17465690

[17] Reeves PG, Chaney RL. Bioavailability as an issue in risk assessment and management of food cadmium: A review. Science of the Total Environment. 2008;398(1–3):13–9. 10.1016/j.scitotenv.2008.03.009 18430461

[18] Kinraide TB. Three mechanisms for the calcium alleviation of mineral toxicities. Plant Physiology. 1998;118(2):513–20. 10.1104/pp.118.2.513 9765536 PMC34826

[19] Zhou X, Hao W, Shi H, Hou Y, Xu Q. Calcium Homeostasis Disruption—a Bridge Connecting Cadmium-Induced Apoptosis, Autophagy and Tumorigenesis. Oncology Research and Treatment. 2015;38(6):311–5. 10.1159/000431032 26045029

[20] Gu J, Ren Z, Zhao J, Peprah FA, Xie Y, Cheng D, et al. Calcimimetic compound NPS R-467 protects against chronic cadmium-induced mouse kidney injury by restoring autophagy process. Ecotoxicology and Environmental Safety. 2020;189. 10.1016/j.ecoenv.2019.110052 31830606

[21] Reeves PG, Chaney RL. Marginal nutritional status of zinc, iron, and calcium increases cadmium retention in the duodenum and other organs of rats fed rice-based. Environmental Research. 2004;96(3):311–22. 10.1016/j.envres.2004.02.013 15364599

[22] Huang F, Wang Z, Zhang J, Du W, Su C, Jiang H, et al. Dietary calcium intake and food sources among Chinese adults in CNTCS. Plos One. 2018;13(10). 10.1371/journal.pone.0205045 30273413 PMC6166981

[23] Saric MM, Blanusa M, Piasek M, Varnai VM, Juresa D, Kostial K. Effect of dietary calcium on cadmium absorption and retention in suckling rats. Biometals. 2002;15(2):175–82. 10.1023/a:1015212929481 12046926

[24] Min K-S, Sano E, Ueda H, Sakazaki F, Yamada K, Takano M, et al. Dietary Deficiency of Calcium and/or Iron, an Age-Related Risk Factor for Renal Accumulation of Cadmium in Mice. Biological & Pharmaceutical Bulletin. 2015;38(10):1557–63. 26228629 10.1248/bpb.b15-00341

[25] Huo Y, Kong X, Liu Y, Zhang J, Wng L, Yang X, inventors; Cangzhou Medical College, assignee. Preparation of sepsis animal model by culturing SPF 8-week-old Balb/c mouse, feeding mice freely into water, alternately illuminating, marking, fixing corresponding serial number, and administering physiological saline, drug and/or bacteria patent CN107982516-A.

[26] Akinyemi AJ, Onyebueke N, Faboya OA, Onikanni SA, Fadaka A, Olayide I. Curcumin inhibits adenosine deaminase and arginase activities in cadmium-induced renal toxicity in rat kidney. Journal of Food and Drug Analysis. 2017;25(2):438–46. 10.1016/j.jfda.2016.06.004 28911688 PMC9332529

[27] Freisinger E VM. Cadmium in metallothioneins. Metal Ions in Life Sciences. 2013;11:339–71. 10.1007/978-94-007-5179-8_11 23430778

[28] Zhang J, Wang Y, Fu L, Wang B, Ji Y-L, Wang H, et al. Chronic cadmium exposure induced hepatic cellular stress and inflammation in aged female mice. Journal of Applied Toxicology. 2019;39(3):498–509. 10.1002/jat.3742 30375035

[29] Wang J, Hao M, Liu C, Liu R. Cadmium induced apoptosis in mouse primary hepatocytes: the role of oxidative stress-mediated ERK pathway activation and the involvement of histone H3 phosphorylation. Rsc Advances. 2015;5(40):31798–806.

[30] Wang Y, Wu Y, Luo K, Liu Y, Zhou M, Yan S, et al. The protective effects of selenium on cadmium-induced oxidative stress and apoptosis via mitochondria pathway in mice kidney. Food and Chemical Toxicology. 2013;58:61–7. 10.1016/j.fct.2013.04.013 23603105

[31] He Q, Luo Y, Zhang P, An C, Zhang A, Li X, et al. Hepatoprotective and antioxidant potential of radish seed aqueous extract on cadmium-induced hepatotoxicity and oxidative stress in mice. Pharmacognosy Magazine. 2019;15(61):283–9.

[32] Brzoska MM, Moniuszko-Jakoniuk J. The influence of calcium content in diet on cumulation and toxicity of cadmium in the organism. Archives of Toxicology. 1998;72(2):63–73. 10.1007/s002040050470 9456077

[33] El-Boshy M, Refaat B, Almaimani RA, Abdelghany AH, Ahmad J, Idris S, et al. Vitamin D-3 and calcium cosupplementation alleviates cadmium hepatotoxicity in the rat: Enhanced antioxidative and anti-inflammatory actions by remodeling cellular calcium pathways. Journal of Biochemical and Molecular Toxicology. 2020;34(3). 10.1002/jbt.22440 31926057

[34] Swarup D, Naresh R, Varshney VP, Balagangatharathilagar M, Kumar P, Nandi D, et al. Changes in plasma hormones profile and liver function in cows naturally exposed to lead and cadmium around different industrial areas. Research in Veterinary Science. 2007;82(1):16–21. 10.1016/j.rvsc.2006.05.002 16822533

[35] Latchoumycandane C, Nagy LE, McIntyre TM. Chronic ethanol ingestion induces oxidative kidney injury through taurine-inhibitable inflammation. Free Radical Biology and Medicine. 2014;69:403–16. 10.1016/j.freeradbiomed.2014.01.001 24412858 PMC3960325

[36] Gong P, Chen F-x, Wang L, Wang J, Jin S, Ma Y-m. Protective effects of blueberries (Vaccinium corymbosum L.) extract against cadmium-induced hepatotoxicity in mice. Environmental Toxicology and Pharmacology. 2014;37(3):1015–27. 10.1016/j.etap.2014.03.017 24751684

[37] Thevenod F, Friedmann JM, Katsen AD, Hauser IA. Up-regulation of multidrug resistance P-glycoprotein via nuclear factor-kappa B activation protects kidney proximal tubule cells from cadmium- and reactive oxygen species-induced apoptosis. Journal of Biological Chemistry. 2000;275(3):1887–96. 10.1074/jbc.275.3.1887 10636889

[38] Kumar N, Kumari V, Ram C, Kumar BSB, Verma S. Impact of oral cadmium intoxication on levels of different essential trace elements and oxidative stress measures in mice: a response to dose. Environmental Science and Pollution Research. 2018;25(6):5401–11. 10.1007/s11356-017-0868-3 29209977

[39] Belcastro M, Marino T, Russo N, Toscano M. The role of glutathione in cadmium ion detoxification: Coordination modes and binding properties—A density functional study. Journal of Inorganic Biochemistry. 2009;103(1):50–7. 10.1016/j.jinorgbio.2008.09.002 18951636

[40] Olisekodiaka MJ, Igbeneghu CA, Onuegbu AJ, Oduru R, Lawal AO. Lipid, Lipoproteins, Total Antioxidant Status and Organ Changes in Rats Administered High Doses of Cadmium Chloride. Medical Principles and Practice. 2012;21(2):156–9. 10.1159/000333385 22076601

[41] Casalino E, Calzaretti G, Sblano C, Landriscina C. Molecular inhibitory mechanisms of antioxidant enzymes in rat liver and kidney by cadmium. Toxicology. 2002;179(1–2):37–50. 10.1016/s0300-483x(02)00245-7 12204541

[42] Zhou Y-j, Zhang S-p, Liu C-w, Cai Y-q. The protection of selenium on ROS mediated-apoptosis by mitochondria dysfunction in cadmium-induced LLC-PK1 cells. Toxicology in Vitro. 2009;23(2):288–94. 10.1016/j.tiv.2008.12.009 19135140

[43] Kagi JHR. OVERVIEW OF METALLOTHIONEIN. Methods in Enzymology. 1991;205:613–26. 10.1016/0076-6879(91)05145-l 1779825

[44] Onosaka S, Min KS, Fukuhara C, Tanaka K. ROLES OF METALLOTHIONEIN IN THE LIVER ON ACUTE CADMIUM TOXICITY IN MICE. 2. EFFECTS OF CALCIUM. Eisei Kagaku-Japanese Journal of Toxicology and Environmental Health. 1983;29(6):407–11.

[45] Washko PW, Cousins RJ. ROLE OF DIETARY CALCIUM AND CALCIUM-BINDING PROTEIN IN CADMIUM TOXICITY IN RATS. Journal of Nutrition. 1977;107(5):920–8. 10.1093/jn/107.5.920 192863

[46] Yamanobe Y, Nagahara N, Matsukawa T, Ito T, Niimori-Kita K, Chiba M, et al. Sex Differences in Shotgun Proteome Analyses for Chronic Oral Intake of Cadmium in Mice. Plos One. 2015;10(3). 10.1371/journal.pone.0121819 25793409 PMC4368563

