# Supplementary material for: Exploration of the optimal strategy for dietary calcium intervention against the toxicity of liver and kidney induced by cadmium in mice: An in vivo diet intervention study
Source: PLoS One. 2021 May 11;16(5):e0250885. doi: 10.1371/journal.pone.0250885 (PMC8112675; doi:10.1371/journal.pone.0250885)
Supplement: S1 Fig — The activity of serum ALT (A) and AST (B) in different groups. (DOCX) [file pone.0250885.s001.docx]

**S1 Fig. The activity of serum ALT(A) and AST(B) in different groups.**
