# Supplementary material for: Exploration of the optimal strategy for dietary calcium intervention against the toxicity of liver and kidney induced by cadmium in mice: An in vivo diet intervention study
Source: PLoS One. 2021 May 11;16(5):e0250885. doi: 10.1371/journal.pone.0250885 (PMC8112675; doi:10.1371/journal.pone.0250885)
Supplement: S5 Fig — (DOCX) [file pone.0250885.s005.docx]

**S5 Fig. The pathological scores of liver and kidney in different groups.**

^*^ P < 0.05, compared with control-group. ^**^ P < 0.01, compared with control-group, using one-way ANOVA.
