# Supplementary material for: Exploration of the optimal strategy for dietary calcium intervention against the toxicity of liver and kidney induced by cadmium in mice: An in vivo diet intervention study
Source: PLoS One. 2021 May 11;16(5):e0250885. doi: 10.1371/journal.pone.0250885 (PMC8112675; doi:10.1371/journal.pone.0250885)
Supplement: S6 Fig — (DOCX) [file pone.0250885.s006.docx]

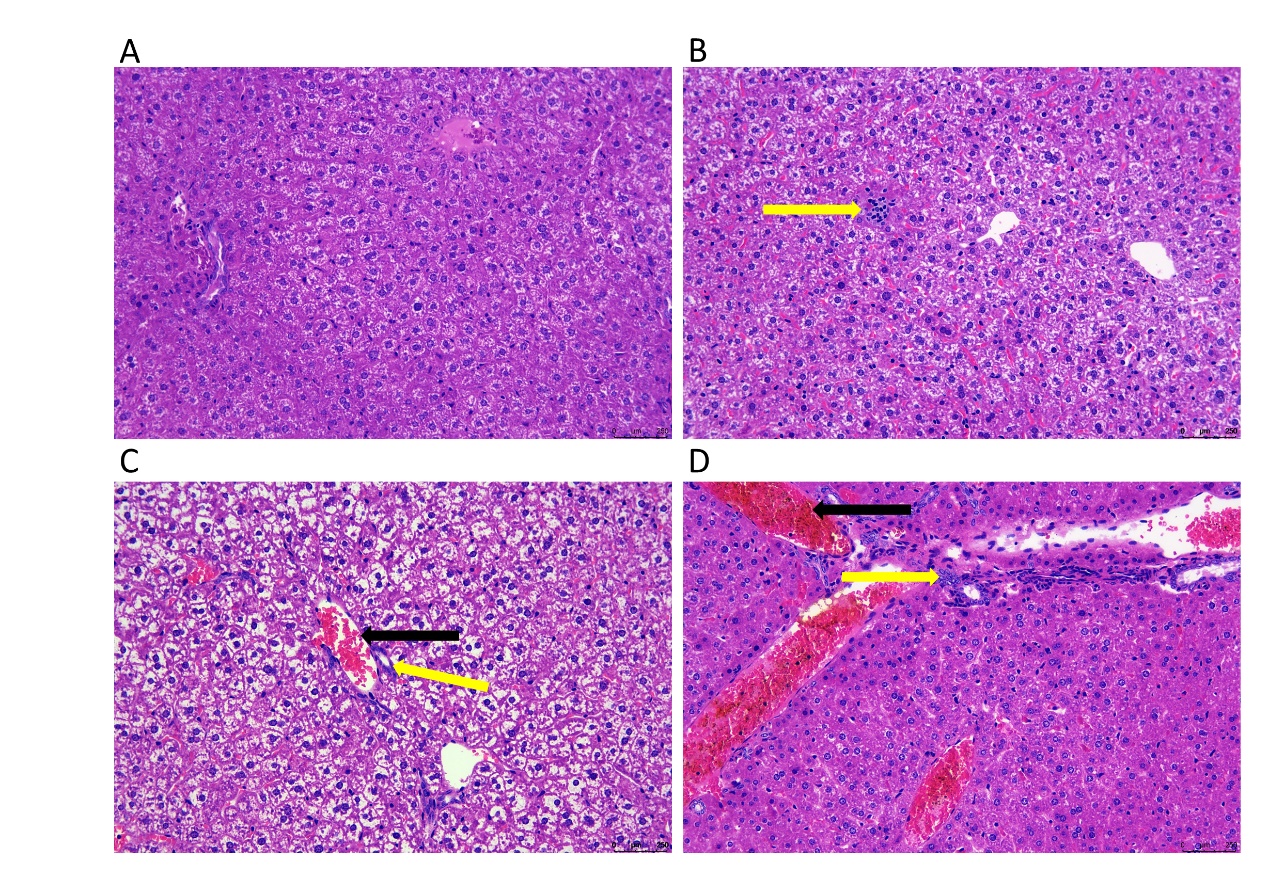


**S6 Fig. The pathological sections of liver in different groups. Pictures were taken at 200×magnification and bar indicates 250 μm.**

A: control-group; B: Cd_L_-group; C: Cd_M_-group; D: Cd_H_-group.

Yellow arrows indicate inflammatory cell infiltration, and black arrows indicate sinus congestion.
