# Supplementary figures and images for: Exploration of the optimal strategy for dietary calcium intervention against the toxicity of liver and kidney induced by cadmium in mice: An in vivo diet intervention study
Source: PLoS One. 2021 May 11;16(5):e0250885. doi: 10.1371/journal.pone.0250885 (PMC8112675; doi:10.1371/journal.pone.0250885)

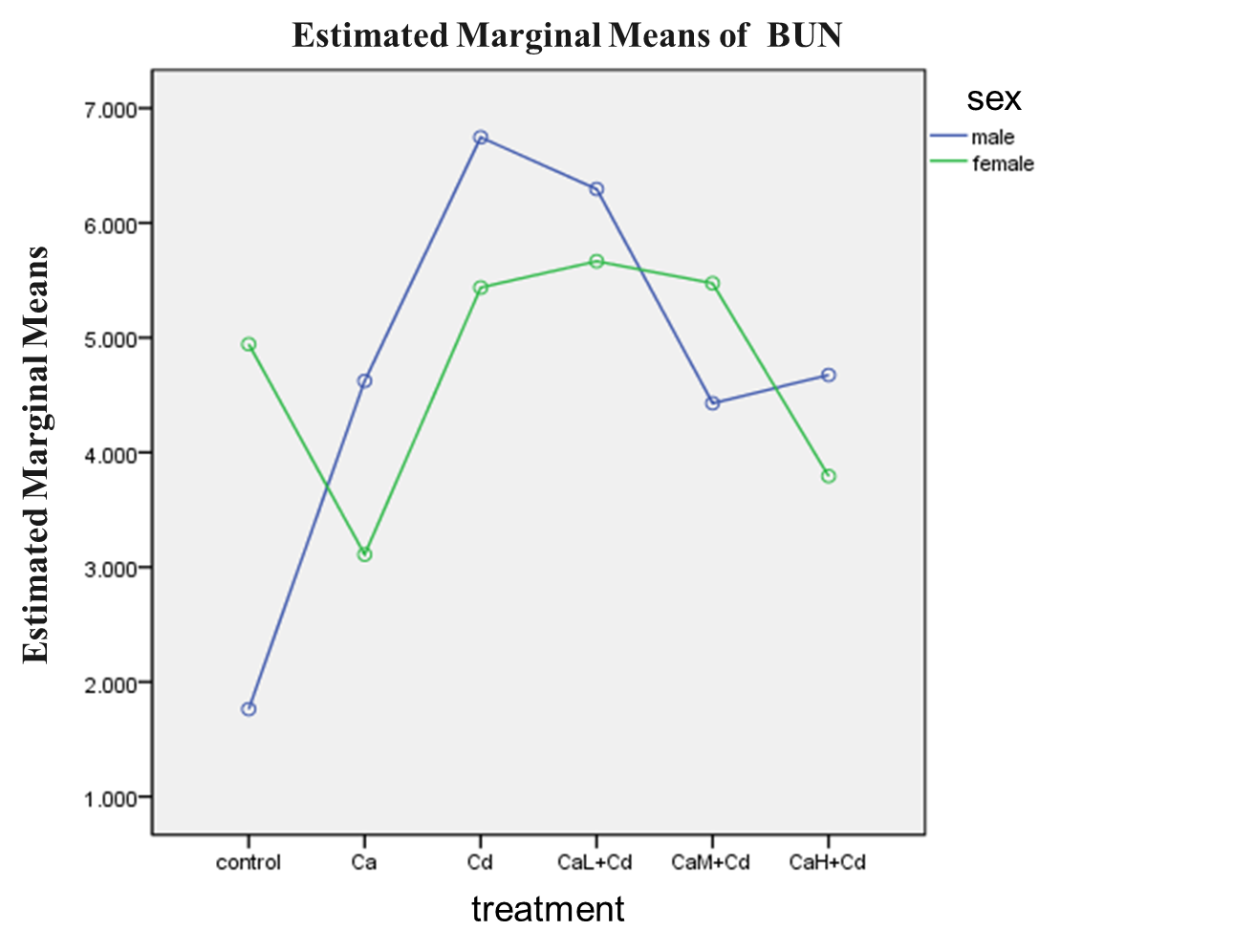


**S8 Fig. The interaction effect of treatments and sexes contributed to serum BUN.**

Supplement: S8 Fig — (DOCX) [file pone.0250885.s008.docx]
