# Supplementary material for: Exploration of the optimal strategy for dietary calcium intervention against the toxicity of liver and kidney induced by cadmium in mice: An in vivo diet intervention study
Source: PLoS One. 2021 May 11;16(5):e0250885. doi: 10.1371/journal.pone.0250885 (PMC8112675; doi:10.1371/journal.pone.0250885)
Supplement: S1 Table — (DOCX) [file pone.0250885.s009.docx]

**S1 Table. Dose of Cadmium in the feed of different experimental groups.**

| Group name | Cd concentration  (mg/kg) |
| --- | --- |
| Control-group | — |
| Cd_L_-group | 0.2 |
| Cd_M_-group | 2 |
| Cd_H_-group | 20 |

Cadmium was added in the mouse feed with the form of Cadmium chloride (CdCl_2_).
