# Supplementary material for: Exploration of the optimal strategy for dietary calcium intervention against the toxicity of liver and kidney induced by cadmium in mice: An in vivo diet intervention study
Source: PLoS One. 2021 May 11;16(5):e0250885. doi: 10.1371/journal.pone.0250885 (PMC8112675; doi:10.1371/journal.pone.0250885)
Supplement: S2 Table — (DOCX) [file pone.0250885.s010.docx]

**S2 Table. Liver and kidney indexes in different groups.**

| Group name | Liver index (%) | Kidney index (%) |
| --- | --- | --- |
| Control-group | 5.53±0.92 | 2.24±0.11 |
| Cd_L_-group | 4.20±0.30^**^ | 2.06±0.17 |
| Cd_M_-group | 4.08±0.71^**^ | 2.05±0.19 |
| Cd_H_-group | 4.53±0.34^*^ | 2.17±0.27 |

Values are expressed as mean ± SD, for 6 animals in each group.

^*^ P < 0.05, compared with control-group. ^**^ P < 0.01, compared with control-group, using one-way ANOVA.
