# Supplementary material for: Exploration of the optimal strategy for dietary calcium intervention against the toxicity of liver and kidney induced by cadmium in mice: An in vivo diet intervention study
Source: PLoS One. 2021 May 11;16(5):e0250885. doi: 10.1371/journal.pone.0250885 (PMC8112675; doi:10.1371/journal.pone.0250885)
Supplement: S3 Table — (DOCX) [file pone.0250885.s011.docx]

**S3 Table. The tests of between-subjects effects in different dependent variables.**

| Dependent variables | Significance | | |
| --- | --- | --- | --- |
|  | treatment | sex | treatment*sex |
| liver index | 0.015* | 0.376 | 0.570 |
| kidney index | 0.001* | 0.000* | 0.468 |
| ALT | 0.000* | 0.624 | 0.209 |
| AST | 0.000* | 0.399 | 0.064 |
| BUN | 0.004* | 0.966 | 0.018* |
| Cr | 0.000* | 0.538 | 0.851 |
| GSH-Px (liver) | 0.000* | 0.066 | 0.771 |
| SOD (liver) | 0.006* | 0.092 | 0.448 |
| CAT (liver) | 0.010* | 0.547 | 0.801 |
| GSH (liver) | 0.000* | 0.131 | 0.195 |
| MDA (liver) | 0.000* | 0.307 | 0.278 |
| GSH-Px (kidney) | 0.000* | 0.576 | 0.055 |
| SOD (kidney) | 0.095 | 0603 | 0.749 |
| CAT (kidney) | 0.000* | 0.265 | 0.162 |
| GSH (kidney) | 0.001* | 0.395 | 0.509 |
| MDA (kidney) | 0.000* | 0.018* | 0.482 |
| liver pathological score | 0.012* | 0.852 | 0.451 |
| kidney pathological score | 0.000* | 0.382 | 0.644 |
| Cd content in liver | 0.000* | 0.168 | 0.115 |
| Cd content in kidney | 0.000* | 0.920 | 0.226 |

Note: * P < 0.05, indicating.
