# Supplementary material for: Exploration of the optimal strategy for dietary calcium intervention against the toxicity of liver and kidney induced by cadmium in mice: An in vivo diet intervention study
Source: PLoS One. 2021 May 11;16(5):e0250885. doi: 10.1371/journal.pone.0250885 (PMC8112675; doi:10.1371/journal.pone.0250885)
Supplement: S4 Table — (DOCX) [file pone.0250885.s012.docx]

**S4 Table. The descriptive statistics of the kidney index and MDA of kidney.**

|  | male | female |
| --- | --- | --- |
| kidney index (%) | 2.303 ± 0.438 | 1.862 ± 0.377 |
| MDA of kidney (nmol/mgprot) | 0.986 ± 0.036 | 0.856 ± 0.036 |

Note: The data are shown as mean ± SD. N=18.
